# The Clinical Outcomes of Ventricular Septal Rupture Secondary to Acute Myocardial Infarction: A Retrospective, Observational Trial

**DOI:** 10.1155/2021/3900269

**Published:** 2021-12-14

**Authors:** Xin-Ying Zhang, Li-Zhao Bian, Nai-Liang Tian

**Affiliations:** Department of Cardiology, Nanjing First Hospital, Nanjing Medical University, Nanjing 210006, China

## Abstract

**Background:**

Ventricular septal rupture (VSR) is a severe mechanical complication secondary to acute myocardial infarction (AMI) with a dreadful prognosis. The goal of our study was to evaluate the mortality and to identify the predictors of mortality for this population.

**Methods:**

From June 2012 to July 2021, patients with VSR secondary to AMI were initially screened for eligibility in this study. The potential risk predictors were determined using appropriate logistic regression models.

**Results:**

In this retrospective study, a total of 50 cases were included, and 14 patients survived and got discharged successfully. Univariable analyses indicated that the heart rate (HR), white blood cell (WBC) count, neutrophils count, serum glucose, serum creatinine, serum lactic acid, and the closure of rupture were significantly associated with mortality among these special populations.

**Conclusion:**

This study found that such high mortality in patients with VSR after AMI was significantly correlated with these risk factors representing sympathetic excitation and large infarct size. Coronary revascularization combined with the closure of rupture might be helpful in improving their prognosis.

## 1. Introduction

Although ventricular septal rupture (VSR) rarely appeared after acute myocardial infarction (AMI), this severe mechanical complication could lead to much higher mortality easily. Currently, the interventional techniques of revascularization had gotten matured gradually, but this mechanical complication was still not eliminated, mainly derived from the unavoidable remodelling based on a large amount of infarcted myocardium [1, 2]. It had been reported that the incidence of VSR after AMI might be 0.17–0.21%, while the mortality could be 41–80% in these subsets [2–5]. In addition, even if these patients survived after this severe complication, they would still be kept in a poor prognosis [6–8]. However, there are still only a few researchers focusing on this topic up to nowadays, mainly because of its rarity. Most of the previous studies were case reports and reported controversial results [9–11]. These conflicting data would seriously limit further explorations for better clinical management of VSR. Therefore, we conducted this present real-world observational study to evaluate the mortality of VSR and try to identify the risk predictors of mortality for these patients.

## 2. Materials and Methods

### 2.1. Patient Selection

All participants in the study were from the Department of Cardiology, Nanjing First Hospital Affiliated with Nanjing Medical University. From June 2012 to July 2021, patients with VSR secondary to AMI were assessed for enrolling in this study, who should meet the followed inclusion criteria: (1) definitely diagnosed as myocardial infarction, including ST-segment elevation myocardial infarction (STEMI) with or without non-ST-segment elevation myocardial infarction (NSTEMI) and (2) with the evidence of left-to-right shunt in ventricular septal based on the ultrasonic cardiogram. AMI was defined as patients with increased cardiac enzymes with myocardial necrosis (including total creatine phosphokinase or creatine kinase major basic fraction >2× the upper limit of the normal range and/or positive troponin I or troponin T) and dynamic changes of the electrocardiogram. Patients with ventricular septal defects secondary to congenital heart disease (CHD) or traumatic cardiac injury should be excluded, even though they had undergone surgical repairment or interventional closure before admission. Moreover, if the onsetting time of AMI was not clear in the medical records, these cases should also be excluded.

### 2.2. Interventional Techniques and Medications

The interventional procedures were performed by 3 experienced interventionists following the current standards. A 300 mg loading dose of clopidogrel and aspirin were routinely applied for these patients before the interventional procedures, and the usage of intraaortic balloon pump (IABP) or other cardiac assistant devices mainly depends on their vital signs. All the implanted stents were drug-eluting stents (DES), and the selection of predilation was decided by these interventionists. Postdilation with noncompliant balloons (≥18 atm pressure) was recommended for all stents (balloon/stent was in a 1 : 1 radio), so that suboptimal expansion or stent mal-apposition might be avoided at all possible. A successful PCI procedure was confirmed when thrombolysis in myocardial infarction (TIMI) grade 3 and residual stenosis <10%. Besides, administering aspirin (100 mg/d) indefinitely and clopidogrel (75 mg/day) for at least 12 months after percutaneous coronary intervention (PCI) was strongly encouraged in these patients. Unfractionated heparin was used for perioperative anticoagulation. In addition, other medications for secondary prevention, including angiotensin-converting enzyme inhibitors, *β*-blockers, or aldosterone antagonists, were appropriately used following the current guidelines [12, 13].

The interventional closure was performed under the guidance of fluoroscopy and echocardiography, as described in a previous report [14]. Amplatzer® ventricular septal defect (VSD) occluders were chosen for these subset populations if necessary. These occluders have been widely used in clinics which are made of two umbrellas and a middle part or “waist,” and polyester fabric over the occluder can help close the holes and provide a foundation for tissue growth after deployment. Most of the included patients underwent interventional closure before PCI. However, 6 patients were diagnosed as complicating VSR after undergoing primary PCI and then received interventional closure. The selection of the closure device was mainly according to the echocardiographic results, which would indicate accurate details of the size and morphology of VSR. At 10 minutes after releasing the occluder, both of angiography and echocardiography were performed to assess the satisfaction for the equipment's position.

### 2.3. Surgery Procedure

The sandwich patch technique through left ventriculostomy has been strongly recommended as standard surgical therapy for VSR secondary to AMI [15, 16]. This advantageous technique could also be achieved by simultaneously performing coronary artery bypass grafting (CABG).

### 2.4. Clinical Data Collection

During the retrospective screening of the medical records, the baseline and procedural characteristics of enrolled patients were collected for further analyses, as well as the relevant laboratory data.

### 2.5. Statistical Analysis

It had been reported that 41–80% of patients were dead from VSR secondary to AMI [2–5]. Accordingly, we estimated the sample size, and a total of 50 patients were finally intended for the enrolment, which would provide 80% power with a 2-sided alpha of 0.05. The baseline characteristics and clinical outcomes were expressed as number, percentage, or mean ± standard deviation (SD) as appropriate. Numerical variables would be shown as median + interquartile range (IQR) values if the data were not normal distribution. The categorical variables in the survival group and the nonsurvival group were compared using Fisher's exact or chi-square test. Student's *t*-test or Wilcoxon rank-sum test was performed for analyzing continuous data as appropriate. The *P* values were 2-tailed, and statistical significance would be confirmed if the *P* value was <0.05. A multivariate binary logistic regression model was used for determining the independent risk predictors for mortality in these patients. All data analyses were performed with SPSS version 22.0 (SPSS Institute, Chicago, USA).

## 3. Results and Discussion

### 3.1. Results

**Figure 1:**
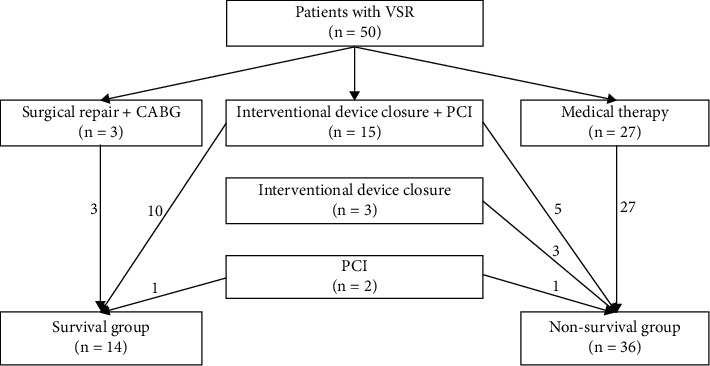
Flowchart for treatment of patients with ventricular septal rupture. VSR, ventricular septal rupture; PCI, percutaneous coronary intervention; CABG, coronary artery bypass grafting.

From June 2012 to July 2021, a total of 50 consecutive patients with VSR secondary to AMI were enrolled in this study. However, only 14 patients survived and got discharged successfully, indicating high mortality of VSR (72%). The baseline characteristics are given in Table 1. Of the 50 enrolled patients, 34.7% of patients were male and 55.1% of patients had diabetes mellitus. Most of the enrolled patients (82%) presented with anterior wall myocardial infarction. Most of the baseline characteristics were generally consistent among the 2 groups and showed no statistical significance. However, better primary cardiac function was observed among patients in the survival group (Killip classification I-II: 78.6% vs. 38.9%, *P* = 0.012) and appeared with much lower heart rate (HR) at admission (94.3 bpm vs. 104.3 bpm, *P* = 0.046). Besides, we stratified these included patients according to the INTERMACS profile [17], showing that patients in the survival group presenting with relatively stable hemodynamic (INTERMACS 1-2: 100% vs. 78.6%, *P* = 0.0186). The procedural characteristics are given in Table 2. There are 22 patients who received coronary angiography (CAG), among whom 59.1% of patients were complicated with multivessel lesions. As shown in Figure 1, there are 20 patients who received interventional treatment in the catheter room, among whom 5 patients underwent either percutaneous coronary intervention (PCI) or interventional closure only. The rest 15 patients received both PCI and interventional closure in sequence. Of these 18 patients who received interventional closure, only 10 patients survived and got discharged, whose survival rate was much lower than that in 3 patients who underwent concomitant CABG during surgical repair (55.6% vs. 100%, *P* < 0.05). However, the whole results indicated that procedural treatment, regardless of interventional or surgical strategies, might lead to better clinical outcomes, as well as the usage of vasoactive drugs.

The laboratory results are given in Table 3. Serum levels of WBC count, neutrophil granulocyte count, serum glucose, serum creatinine, and serum lactic acid showed statistically significant differences between the two groups, while other parameters indicated no statistical significance.

Based on univariate analysis, seven univariables (*P* < 0.1), including HR, were correlated with mortality among these special subsets. To confirm independent risk predictors of mortality in VSR patients and to avoid overadjustment and collinearity, HR was adjusted with serum WBC count and glucose because they were mismatched in the two groups, indicating statistical insignificance under multivariate analysis (Table 4).

### 3.2. Discussion

This present retrospective, observational study indicated much high mortality in VSR after AMI. Moreover, these risk factors representing sympathetic excitation and larger infarcted myocardium showed markable correlations with mortality. Significant reductions in death would be achieved when procedural treatment, regardless of interventional or surgical strategies, was performed as soon as possible.

It had been widely noted that VSR was a rare mechanical complication of AMI but could lead to much high mortality. The present study reported that the mortality of VSR after AMI was 72%, which was in line with that in the SHOCK trial [18]. Furthermore, our study identified several predictors with respect to the incidence of death.

HR, considered as the most intuitive index of sympathetic excitation, showed a significant association with mortality in these patients. In the past several decades, it has been noted that lower HR could significantly decrease myocardial oxygen demand and metabolic requirements, which might help improve the clinical outcomes [19]. Similarly, results from this study demonstrated the benefits of lowering HR in these subsets because it could also represent fewer infarcted myocardium and better cardiac function.

Of note, leukocyte plays an important role in systemic inflammatory reactions, and AMI is commonly accompanied by a severe inflammatory response. Cannon et al. had reported that significantly increased WBC count (>10,000) was associated with increased 30-day and 10-month mortality in patients with acute coronary syndrome [20]. Bradley et al. indicated that the elevated leukocyte count was an independent risk factor with respect to in-hospital mortality of AMI [21]. Therefore, risk stratification based on leukocyte count could not be ignored, and dynamic monitoring of the parameters seemed to be necessary for guiding the clinical decision making for managing these VSR patients.

On the other hand, the serum glucose level was also associated with mortality according to multivariable logistic regression. In this study, the serum glucose level was much higher in the nonsurvival group. Although this result was not described previously in these VSR patients, it might be explained by the stress-related hyperglycemia induced by AMI. In fact, several prior studies confirmed that hyperglycemia stress could be considered as an independent risk factor for acute kidney injury (AKI) and adverse cardiovascular events in AMI patients without diabetes [22, 23]. Therefore, the beneficial impact on improving the prognosis of VSR patients via reducing the hyperglycemia stress should also draw attention. More powerful related trials are still warranted.

Besides, the importance of the closure of VSR secondary to AMI should also be noted. Sabiniewicz et al. had reported much higher mortality if patients were treated with conservative medical therapy alone [24]. Similarly, we also found that the mortality from medical management alone was much higher than that in patients receiving procedural treatment (100% vs. 45%, *P* < 0.05). Nowadays, interventional closure for VSR has obtained more scholarly attention as showing better clinical results, especially for those with hemodynamic instability, indicating higher surgical risk [14, 15]. One recent study showed non-inferior effects of interventional closure in reducing mortality when compared with surgical repair (54% vs. 56%, *P* = 0.82) [25]. Thus, both surgical repair and interventional closure should be recommended to reduce mortality and to improve prognosis.

### 3.3. Limitations

There were several limitations in the present study. First, this is a retrospective, observational study. There might be some potential bias from the collection of nonrandomized data, missing or incomplete information to sway the final results. Second, the small sample size meant that it was not possible to demonstrate all risk factors associated with the mortality of VSR. Third, although the whole results occurred in the hospital, it still might be a better choice to extend the clinical follow-up for assessing long-term clinical outcomes. In addition, different types of implanted stents and closure devices or surgical procedures also limited us to study the true benefits of procedural treatment. Finally, several other risk factors, including the adjustment of medication regimen, compliance to drugs, should also be considered as important interfering factors.

## 4. Conclusions

This present retrospective, observational study showed much high mortality in VSR after AMI. As expected, these risk factors representing sympathetic excitation and larger infarcted myocardium were demonstrated to have strong associations with mortality in these subsets. Procedural treatments, including primary PCI and interventional closure or surgical repair, were strongly recommended for better clinical outcomes, as well as the appropriate clinical management of the general status. These findings might provide some advanced suggestions for clinical management of these VSR patients secondary to AMI.

## Figures and Tables

**Table 1:** Baseline characteristics of patients with ventricular septal rupture.

Parameter	Nonsurvival group	Survival group	*P* value
	(*n* = 36)	(*n* = 14)	
Age, mean (SD), *y*	72.3 (8.6)	68.7 (6.9)	0.176
Male gender, *n* (%)	12 (33.3)	6 (42.9)	0.529
Diabetes mellitus, *n* (%)	22 (61.1)	6 (42.9)	0.243
Hypertension, *n* (%)	29 (80.5)	9 (64.3)	0.278
Hyperlipidemia, *n* (%)	21 (58.3)	9 (64.3)	0.700
History of stroke, *n* (%)	8 (22.2)	2 (14.3)	1.000
Positive smoking status, *n* (%)	13 (36.1)	6 (42.9)	0.659
Pulmonary infection, *n* (%)	13 (36.1)	2(14.3)	0.179
Pericardial effusion, *n* (%)	6 (16.7)	0	0.167
Cardiac arrhythmia, *n* (%)	7 (19.4)	0	0.169
Renal failure, *n* (%)	3 (8.3)	3 (21.4)	0.331
Liver failure, *n* (%)	5 (13.9)	1 (7.1)	0.663
MODS, *n* (%)	5 (13.9)	0	0.304
Cardiac and respiratory arrest, *n* (%)	4 (11.1)	1 (7.1)	0.176
AMI of anterior wall, *n* (%)	31 (86.1)	10 (71.4)	0.245
AMI of nonanterior, *n* (%)	5 (13.9)	4 (28.6)	
Multisite of AMI (percent, %)	11(30.6)	1 (7.1)	0.140
Killip classification I-II, *n* (%)	**14 (38.9)**	**11 (78.6)**	**0.012**
Killip classification III-IV, *n* (%)	**22 (61.1)**	**3 (21.4)**	
HR, mean (SD), bpm	**104.3 (15.0)**	**94.3 (16.0)**	**0.046**
SBP, mean (SD), mmHg	109.3 (17.1)	119.2 (34.6)	0.320
DBP, mean (SD), mmHg	72.5 (12.0)	78.4 (20.0)	0.203
LVEF, mean (SD), *n* (%)	49.6 (9.9)	47.50 (8.7)	0.616
Erythrocyte substitution, *n* (%)	6 (16.7)	5 (28.6)	0.252
Thrombocyte substitution, *n* (%)	2 (5.6)	1 (7.1)	1.000
Fresh frozen plasma, *n* (%)	3 (8.3)	0	0.550
IABP, *n* (%)	35 (97.2)	11 (78.6)	0.101
CRRT, *n* (%)	4 (11.1)	0	0.201
Ventilator, *n* (%)	8 (22.2)	0	0.087
Vasoactive drugs, *n* (%)	15 (41.7)	5 (35.7)	0.700
CPR, *n* (%)	5 (13.9)	0	0.304
INTERMACS 1-2	**36 (100)**	**11 (78.6)**	**0.019**
INTERMACS 3–5	**0**	**3 (21.4)**	

MODS, multiple organ dysfunction syndrome; AMI, acute myocardial infarction; HR, heart rate; bpm, beat per minute; SBP, systolic blood pressure; DBP, diastolic blood pressure; LVEF, left ventricle eject fraction; IABP, intraaortic balloon pump; CRRT, continuous renal replacement therapy; CPR, cardiopulmonary resuscitation, INTERMACS profile, The Interagency Registry for Mechanically Assisted Circulatory Support profile. All the values in bold are associated with a statistical difference (*P* < 0.05).

**Table 2:** Procedural characteristics of patients with ventricular septal rupture.

Parameter	Nonsurvival group	Survival group	*P* value
	(*n* = 36)	(*n* = 14)	
The closure of VSR (surgical repair or device closure), *n* (%)	**8 (22.2)**	**13 (92.9)**	**<0.001**
Surgical repair, *n* (%)	**0**	**3 (21.4)**	**0.019**
Device closure, *n* (%)	**8 (22.2)**	**10 (71.4)**	**0.003**
Reperfusion, *n* (%)	**6 (16.7)**	**14 (100)**	**<0.001**
PCI, *n* (%)	**6 (16.7)**	**11 (78.6)**	**0.001**
CABG, *n* (%)	**0**	**3 (21.4)**	**0.019**
Medical therapy, *n* (%)	**27 (75)**	**0**	**<0.001**
Number of lesioned vessels			
Triple vessels, *n* (%)	**3 (37.5)**	**10 (71.4)**	**0.022**
Double vessels, *n* (%)	**2 (25)**	**4 (28.6)**	
Single vessel, *n* (%)	**3 (37.5)**	**0**	
Diameter of rupture, mean (SD), mm	13.9 (4.9)	14.1 (5.0)	0.248
Qp/Qs, mean (SD)	4.3 (2.5)	3.9 (1.6)	0.793
The mean duration from AMI to VSR, median (IQR), days	1.5 (0–3.25)	1.5 (0–3.0)	0.443
The mean duration from the onset of AMI to closure of VSR, median, (IQR), days	14.88 (13.5–20.0)	18.00 (14.0–22.0)	0.345
The mean duration from AMI to reperfusion, median, (IQR), days	18.08 (14.0–23.54)	24.50 (22.3–30.25)	0.390

PCI, percutaneous coronary intervention; CABG, coronary artery bypass grafting; Qp, pulmonary blood flow; Qs, systemic blood flow;AMI, acute myocardial infarction. A number of 22 patients underwent coronary angiography during hospitalization. IQR, interquartile range. All the values in bold are associated with a statistical difference (*P* < 0.05)

**Table 3:** Laboratory data of patients with ventricular septal rupture.

Parameter	Nonsurvival group	Survival group	*P* value
	(*n* = 36)	(*n* = 14)	
Hemoglobin, mean (SD), G/l	125.7(21.1)	115.50(14.5)	0.560
WBC count, G/l	**19.71 (5.982)**	**13.12 (2.892)**	**<0.001**
Neutrophil count, G/l	**17.48 (5.45)**	**10.77 (3.246)**	**< 0.001**
Neutrophilic percentage	86.15 (5.533)	84.96 (5.646)	0.520
Lymphocyte count, G/l	1.98 (2.006)	1.31 (0.565)	0.390
NLR, %	18.96 (22.766)	10.03 (4.246)	0.314
Percentage of lymphocytes, %	7.96 (4.796)	9.87 (4.707)	0.353
PLT count, G/l	171.26 (97.842)	241.46 (115.211)	0.052
CK, U/L	1223.3(1232.5)	761.5 (725.2)	0.609
CK-MB, U/l	132.4 (258.1)	84.1 (104.5)	0.350
AST, U/l	1832.4 (2694.9)	393.0 (820.0)	0.058
ALT, U/l	976.6(1578.5)	311.7 (417.8)	0.182
CTn I, ng/ml	100 (396.6)	13.2 (20.2)	0.498
CTn T, ng/ml	2863.7 (2838.7)	1043.1 (740.3)	0.057
PLA_2_, ng/ml	340.7 (156.3)	265.0 (105.3)	0.273
Myohemoglobin, ng/ml	227.1 (116.4)	120.7 (146.5)	0.129
NT-BNP, pg/l	18414.7 (12049.4)	10752.0 (12533.2)	0.109
TG, mmol/l	2.54 (1.240)	1.93 (0.713)	0.223
TC, mmol/l	3.7 (1.4)	3.4 (1.0)	0.602
LDL, mmol/l	3.2 (0.9)	2.5 (0.7)	0.129
SCr, umol/l	**237.8 (184.2)**	**123.1 (60.0)**	**0.035**
Serum lactic acid, mmol/l	**6.0 (4.0)**	**2.2 (0.8)**	**0.019**
Serum glucose, mmol/l	**16.9 (5.9)**	**12.2 (4.0)**	**0.012**
HbA1c, %	8.0 (2.0)	6.4 (0.7)	0.095
Serum uric acid, umol/l	684.7 (193.3)	525.1(156.0)	0.057

WBC, white blood cell; NLR, neutrophil to lymphocyte ratio; AST, aspartate aminotransferase; ALT, alanine aminotransferase; CTn I, cardiac troponin I; CTn T, cardiac troponin T; PLA2, lipoprotein-associated phospholipase A2; NT-BNP, N-terminal prohormone of brain natriuretic peptide; TG, triglyceride; TC, total cholesterol; LDL, low-density lipoprotein, SCr, serum creatinine; HbA1c, glycated hemoglobin. All the values in bold are associated with a statistical difference (*P* < 0.05)

**Table 4:** Univariable and multivariables analysis predicating survival in hospital.

Predictor	Univariables OR (95% CI)	*P* value	Multivariables OR (95% CI)	*P* value
Killip class	0.514 (0.134–1.98)	0.333		
HR	**0.956 (0.913–1.000)**	**0.053**		
The closure of rupture	**45.5 (5.141–402.685)**	**<0 .001**		
Number of lesion coronary artery				
Two-vessel lesion	3.0 (0.312–28.84)	0.341		
Three-vessel lesion	9.0 (0.563–143.89)	0.120		
WBC count	**0.736 (0.598–0.906)**	**0.004**	**0.776 (0.604–0.998)**	**0.048**
Neutrophil count	**0.658 (0.503–0.860)**	**0.002**		
SCr	**0.990 (0.981–0.999)**	**0.036**	0.993 (0.978–1.008)	0.361
Serum lactic acid	**0.285 (0.0796–1.02)**	**0.054**		
Serum glucose	**0.797 (0.658–0.965)**	**0.020**	**0.751 (0.568–0.992)**	**0.044**

HR, heart rate; WBC, white blood cell; SCr, serum creatinine. The values in bold are related to a significant difference (*P* < 0.10) in the univariable logistic regression model and a significant difference (*P* < 0.05) in the multivariable regression model.

## Data Availability

The clinical data used to support the findings of this study are available from the corresponding author upon request.
